# Benchmarking AI-Powered Translation of the EQ-5D-5L Patient-Reported Outcome Measure Using Automated Metrics: Comparative Evaluation Study

**DOI:** 10.2196/78485

**Published:** 2026-08-04

**Authors:** Himanshu Vashisht, Tomás Ward, Willie Muehlhausen

**Affiliations:** 1 School of Computing, Dublin City University, Collins Ave Ext, Whitehall, Dublin, Ireland, 353 899586131; 2 Rinn Artificial Intelligence, Dublin City University, Dublin, Ireland; 3 SAFIRA Clinical Research Ltd, Tipperary, Ireland

**Keywords:** patient-reported outcomes, electronic clinical outcome assessment, machine translation, EQ-5D-5L, translation quality, linguistic validation, clinical research, multilingual studies

## Abstract

**Background:**

Patient-reported outcome measures (PROMs) are central to multinational clinical research, but high-quality translation and linguistic validation remain resource-intensive. AI-powered translation may accelerate this process, but its performance relative to validated human PROM translations requires systematic evaluation.

**Objective:**

This benchmarking study evaluated the quality and comparability of 4 AI-powered translation services for the EuroQol 5-dimension 5-level (EQ-5D-5L) across 5 target languages, using official, linguistically validated human translations as the reference standard (gold standard).

**Methods:**

The 43 text segments of the EQ-5D-5L were translated from English into Danish, Dutch, French, German, and Spanish using Google Translate, GPT-4.1, Amazon Translate, and DeepL. GPT-4.1 was evaluated with a structured medical-translator prompt, whereas Google Translate, Amazon Translate, and DeepL were evaluated using standard unprompted application programming interfaces without domain-specific glossary constraints. Outputs were benchmarked against official, validated human translations using 4 automated metrics: BLEU (bilingual evaluation understudy), METEOR (metric for evaluation of translation with explicit ordering), COMET (cross-lingual optimized metric for evaluation of translation), and BLEURT (bilingual evaluation understudy with representations from transformers). Friedman tests were used to assess overall between-service differences within each metric-language combination. When the Friedman test was significant, paired Wilcoxon signed-rank post hoc tests with Holm-Bonferroni correction were conducted. Descriptive summaries, score distributions, and sentence-level hotspot analyses were used to evaluate semantic similarity patterns and identify localized low-scoring deviations.

**Results:**

Friedman tests assessed whether the AI services differed in performance, whereas descriptive summaries and visualizations were used to determine whether scores clustered in ranges consistent with strong semantic similarity to the gold standard. Friedman tests identified statistically significant between-service differences in 11 of the 20 (55%; *P*<.05) metric-language combinations. Subsequent paired Wilcoxon signed-rank post hoc tests with Holm-Bonferroni correction identified 11 significant pairwise differences, with adjusted *P* values ranging from <.001 to .049. Most of these differences were detected by surface-overlap metrics (10/11 for BLEU or METEOR), whereas only 1 of 11 was detected by a semantic metric (BLEURT), suggesting that many between-service differences were stylistic rather than meaning-altering. Descriptive and visual analyses further showed that semantic similarity was generally high across services, while low-scoring deviations clustered in specific linguistic hotspots, particularly domain headers, abstract health concepts, and short, context-dependent interface strings.

**Conclusions:**

Among the evaluated high-resource European languages, AI translation services showed high semantic similarity to the validated human translations, although localized conceptual deviations persisted. These findings suggest that AI can support the generation of translations for PROM workflows in these languages; however, expert human review may still be required to confirm conceptual equivalence. The practical relevance of isolated header differences could not be assessed in the present study, whereas abstract health concepts and other clinically sensitive phrasing should be evaluated in further research.

## Introduction

### Background

Patient-reported outcome measures (PROMs) are pivotal in clinical research because they capture health status, symptoms, functioning, and quality of life directly from patients. The increasing global reach of clinical trials necessitates that PROMs be accurately translated and culturally adapted for diverse linguistic and cultural contexts. Traditionally, this process has involved a rigorous linguistic validation methodology, typically including forward and backward-translation, cognitive debriefing, and expert review, ensuring conceptual, item, and measurement equivalence across languages [1]. This comprehensive approach, while robust, is resource-intensive and time-consuming, posing challenges for the efficient deployment of PROMs in multinational studies [2,3].

### Challenges in Traditional Translation

The traditional linguistic validation process, while crucial for ensuring high-quality PROM translations, faces several inherent challenges. These include the significant time and financial resources required to execute rigorous forward-backward translation, cognitive debriefing, and expert reconciliation processes [4]. The complexity of managing these multistep workflows across numerous languages can lead to delays in clinical trial timelines, impacting the overall efficiency and cost-effectiveness of global research [5]. Furthermore, the availability of qualified human translators with expertise in both medical terminology and specific cultural nuances can be limited, particularly for less commonly spoken languages or highly specialized therapeutic areas [6].

### Emergence of AI in Translation

The rapid advancements in AI, particularly in natural language processing and, more recently, large language models (LLMs), have introduced new possibilities for automated and semiautomated translation. AI-powered translation services offer the potential to significantly streamline the translation process, reduce costs, and accelerate the availability of PROMs in multiple languages [7,8]. These technologies leverage vast datasets and sophisticated algorithms to generate translations with remarkable fluency and, in many cases, accuracy comparable to that of human translation for general texts [9,10]. The integration of AI tools could potentially alleviate some of the burdens associated with traditional translation workflows, making multilingual electronic clinical outcome assessment deployment more feasible and efficient [11,12]. Recent PROM-specific evidence also suggests that LLMs can produce clinically relevant translation outputs under certain conditions, although performance remains dependent on the instrument, language pair, and evaluation framework [13]. However, recent health care research has emphasized that LLMs should be evaluated and deployed with careful attention to oversight, transparency, and task-specific risk, particularly in clinically consequential settings [14].

### Overview of AI Translation Models

Current AI translation tools vary in architecture and controllability. Google Translate, Amazon Translate, and DeepL are widely used neural machine translation (NMT) services, whereas GPT-4.1 represents a newer LLM-based approach that can be guided through explicit prompting [9,10,15-17]. Understanding how these systems compare with industry-standard clinical translations (linguistic validation) is important before they can be used responsibly in sensitive settings such as PROM translation. Recent research suggests that machine translation in the era of LLMs has improved substantially, especially in high-resource languages, while still exhibiting limitations that may remain relevant for specialized applications such as clinical questionnaires [18].

### Specific Characteristics of EQ-5D-5L for Translation

The EuroQol 5-dimension 5-level (EQ-5D-5L) is a widely used generic PROM for assessing health-related quality of life across various populations and diseases [19]. Its standardized, simple, and direct language structure, consisting of 5 dimensions (mobility, self-care, usual activities, pain or discomfort, and anxiety or depression), each with 5 severity levels, makes it seem straightforward to translate. However, ensuring the linguistic integrity and measurement equivalence of its translations is critical for the validity of data collected in global trials [20,21]. Ensuring conceptual equivalence is, therefore, essential to preserve the instrument’s validity in multinational research.

### Goal of This Study

This study aimed to benchmark (via algorithms) the quality and comparability of AI-generated EQ-5D-5L translations against linguistically validated human translations. It was designed as a practical benchmarking study against an established clinical translation standard rather than as a test of full linguistic validation or zero-shot performance on unseen proprietary text.

## Methods

### Study Design

**Figure 1.**
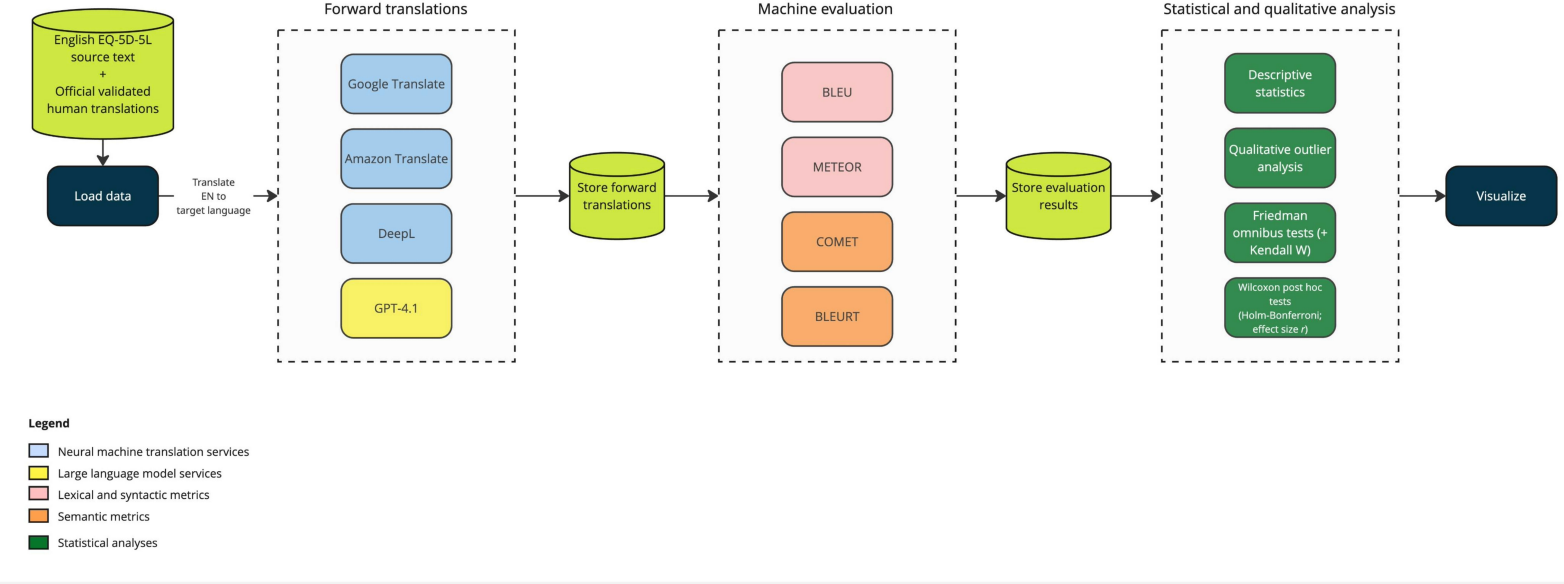
Study workflow for this methodological benchmarking study of AI-powered EQ-5D-5L translation. The 43 UK English EQ-5D-5L source segments were translated into 5 target languages (Danish, Dutch, French, German, and Spanish) using 4 AI services (Google Translate, Amazon Translate, DeepL, and GPT-4.1). AI-generated forward translations were then benchmarked against official, linguistically validated human translations using 4 automated metrics (BLEU, METEOR, COMET, and BLEURT). The resulting scores were summarized using descriptive statistics, qualitative outlier analysis, and inferential testing (Friedman tests with Kendall *W* and Holm-Bonferroni-adjusted Wilcoxon post hoc comparisons), followed by visualization of score distributions and hotspot patterns. BLEU: bilingual evaluation understudy; BLEURT: bilingual evaluation understudy with representations from transformers; COMET: cross-lingual optimized metric for evaluation of translation; EN: English; EQ-5D-5L: EuroQol 5-dimension 5-level; METEOR: metric for evaluation of translation with explicit ordering.

This study used a comparative benchmarking design to assess the quality and comparability of AI-powered translation outputs of the EQ-5D-5L against official, linguistically validated human translations. The methodology involved translating the standardized English version of the EQ-5D-5L into 5 target high-resource target languages: Danish (DA), Dutch (NL), French (FR), German (DE), and Spanish (ES), using 4 distinct AI translation services. Figure 1 illustrates the comprehensive workflow used in this study, from the initial document translation to the final quality evaluation and analysis.

### Translation Services Used

Four leading AI translation services were selected for this study:

- Google Translate: This is a widely used NMT service known for its extensive language support and continuous advancements [10].
- GPT-4.1 (OpenAI): This is a state-of-the-art LLM [17]. For this study, translations were generated through the OpenAI application programming interface (API) using the GPT-4.1 snapshot identifier, gpt-4.1-2025-04-14. To ensure reproducibility, fixed settings of a seed of 42 and a temperature of 0 were used. A detailed system prompt was used to instruct the model to act as a professional medical translator, emphasizing accuracy, clinical context, and fidelity to the source text (see Multimedia Appendix 1 for the full prompt). No equivalent prompt-based configuration was available in the standard workflows used by the NMT services.
- Amazon Translate (Amazon Web Services [AWS]): This is an NMT service offering high scalability and integration with other cloud services [16].
- DeepL: This is an NMT service renowned for its high-quality, nuanced translations, particularly for European languages, leveraging advanced deep learning architectures [15].

These services represent a diverse set of current AI translation technologies. No equivalent domain-specific prompt, medical glossary, or custom terminology constraint was applied to Google Translate, Amazon Translate, or DeepL. These services were evaluated using their standard API translation behavior, as commercially available to end users at the time of analysis. Because the evaluated systems differ in interface and controllability, input standardization was necessarily imperfect. GPT-4.1 permitted explicit instruction through a system prompt, whereas the NMT services did not provide an equivalent prompt-based mechanism within the workflow used here. Therefore, this study reflects a realistic applied comparison of available translation services rather than a strictly parameter-matched head-to-head experiment.

### Provenance and Reproducibility

To address potential model drift and ensure reproducibility, all AI translations were generated within a fixed time window in August 2025. The specific service versions and end points used were as follows:

- Google Translate: Requests were submitted through the Google Cloud Translation API (version 3) end point
- DeepL: Requests were made to the DeepL API (version 2) end point
- Amazon Translate: Requests were made through the AWS software development kit for Python (boto3; version 1.40.66), targeting the eu-west-1 region
- GPT-4.1: As detailed previously, all requests used the GPT-4.1 API snapshot gpt-4.1-2025-04-14, with a fixed seed of 42 and a temperature of 0

The access dates reported for API documentation references indicate when the documentation sources were consulted and do not necessarily correspond to the dates on which translation outputs were generated.

### Data Collection and Preparation

The source text consisted of the 43 unique text segments from the standard UK English version of the EQ-5D-5L, comprising the instrument’s 5 dimensions and the EQ visual analog scale instructions. For interpretive transparency, Multimedia Appendix 2 presents annotated screenshots of the corresponding sample UK English digital questionnaire structure from the EuroQol user guide. These text segments were systematically translated into the 5 target languages using each of the 4 AI translation services. Official, linguistically validated human translations for each target language were obtained from the EuroQol Research Foundation and served as the gold standard reference for benchmarking. These translations were developed through a multistep process, including forward-backward translation and cognitive debriefing, and represent the current industry standard for clinical use.

Sentence-level translation was used because the workflow compared each source segment with its aligned gold standard counterpart, and many EQ-5D-5L elements function as discrete response or instruction units rather than cohesive prose. However, this design removed the broader questionnaire context that some systems might otherwise use to improve lexical consistency, disambiguation, and register, and it provided only an approximation of dependence among related instrument segments.

### Automated Translation Quality Metrics

Evaluating the quality of machine-translated PROMs requires robust and reliable metrics. This study uses 4 widely recognized automated machine translation metrics. All scores were computed at the sentence level.

- BLEU (bilingual evaluation understudy): BLEU is a precision-based metric that measures *n*-gram (contiguous sequences of *n* words) overlap between the candidate and reference translations [22]. We used sacrebleu (version 2.5.0) with the “13a” tokenizer and normalized scoring. BLEU scores range from 0 to 1, with 1 indicating a perfect match to the reference. Generally, BLEU scores >0.5 reflect high-quality, fluent translations, whereas scores <0.3 often indicate poor correlation with human references.
- METEOR (metric for evaluation of translation with explicit ordering): METEOR is a recall-oriented metric that considers unigram (single word) matching, stemming, and synonym matching [23]. Scores were computed using Natural Language Toolkit (version 3.9.1). METEOR scores range from 0 to 1, with higher scores indicating better translation quality. Similar to BLEU, scores approaching 1.0 indicate high quality, whereas lower scores suggest significant lexical deviation.
- COMET (cross-lingual optimization for machine translation evaluation): COMET is a neural framework that uses a pretrained cross-lingual encoder to produce more robust evaluations by assessing semantic similarity [24]. We used the Unbabel/wmt22-comet-da reference-based model, which has shown a high correlation with human judgments. COMET scores typically range from −1 to 1, with 1 representing a perfect translation. While no universal threshold exists, scores ≥0.80 typically indicate strong semantic equivalence, whereas scores <0.60 often signal potential semantic errors requiring review.
- BLEURT (bilingual evaluation understudy representation-enhanced): BLEURT is a neural metric trained to predict human judgments of translation quality [25]. We used the official BLEURT-20 checkpoint. BLEURT scores generally range from −1 to 1, with higher scores indicating better quality. Consistent with COMET, scores approaching 1.0 reflect high semantic fidelity.

BLEU and METEOR are more sensitive to lexical and word-order variation, whereas COMET and BLEURT are more informative for semantic adequacy. Because COMET and BLEURT were used in a reference-based form against the gold standard translations, they may reward closer alignment with the validated reference wording and penalize clinically acceptable alternative phrasings. Recent work has also aimed to improve the interpretability of neural translation metrics through fine-grained error detection, reinforcing the importance of semantic evaluation approaches that move beyond simple lexical overlap [26].

To aid interpretation, we also summarized the proportion of sentence-level scores falling within descriptive quality bands. Because no universally accepted pass or fail thresholds exist for applying these automated metrics to PROM translation benchmarking, the bands were used only as pragmatic interpretive aids for summarizing score distributions. For BLEU and METEOR, which are more sensitive to lexical and word-order overlap, scores ≥0.50 were categorized as high, scores from 0.30 to <0.50 as mid, and scores <0.30 as low. For COMET and BLEURT, which were used as semantic similarity metrics, scores ≥0.80 were categorized as high, scores from 0.60 to <0.80 as mid, and scores <0.60 as low. These bands were not treated as formally validated pass or fail criteria for PROM translation quality. These metrics provide objective, quantitative measures of translation quality that are crucial for benchmarking different AI services.

### Data Analysis

#### Overview of Analyses

The analyses addressed 2 complementary questions. First, inferential comparisons were used to test whether the 4 AI services differed in performance within each metric-language combination. Second, descriptive summaries, violin plots, and sentence-level heatmaps were used to evaluate whether outputs were generally concentrated in ranges consistent with acceptable semantic similarity to the validated reference translations.

#### Descriptive Statistics

For each AI service within each metric-language combination, we calculated the median and IQR across the 43 source segments to summarize the central tendency and dispersion of sentence-level translation quality scores.

#### Inferential Statistics and Effect Sizes

Given the repeated-measures design, Friedman tests were used to compare the score distributions among the 4 AI services within each metric-language combination. To quantify the magnitude of any significant findings, Kendall *W* was calculated as the omnibus effect size. When the Friedman test was significant (*P*<.05), paired Wilcoxon signed-rank post hoc tests were performed with Holm-Bonferroni correction, and the pairwise effect size *r* was calculated for each significant contrast. Because the Friedman and Wilcoxon signed-rank post hoc tests address different hypotheses, omnibus Friedman *P* values were interpreted separately from Holm-Bonferroni–adjusted pairwise *P* values. Kendall *W* and the pairwise Wilcoxon effect size *r* quantify different levels of comparison and should not be interpreted as directly interchangeable. Kendall *W* summarizes the overall degree of separation among all 4 services within a metric-language combination, whereas *r* quantifies the magnitude of a specific pairwise contrast.

#### Qualitative Outlier Analysis

Finally, to supplement the inferential and descriptive analyses, a qualitative outlier analysis was conducted to identify localized failure modes. For each metric-language combination, sentence-level scores at or below the 5th percentile were flagged as potential hotspots and aggregated across services, languages, and metrics to identify recurring low-scoring segments. As a descriptive sensitivity analysis, we also summarized the total number of flagged instances generated by each metric across all language-service combinations to compare how frequently each metric identified potential problem segments. This comparison was descriptive and was intended to characterize relative filtering sensitivity rather than to provide a separate inferential test.

Flagged discrepancies were then reviewed qualitatively and assigned to prespecified linguistic error categories. Conceptual errors referred to translations that failed to convey the intended clinical construct represented in the gold standard. Semantic errors referred to meaning shifts that altered the sense of the item without necessarily changing its conceptual domain. Intensity errors referred to translations that distorted the severity or strength of the source wording. Domain errors referred to translations that shifted a term into an inappropriate contextual field or usage domain. These categories were used as an interpretive framework for describing representative outliers rather than as a formally validated taxonomy. Categorization was informed by comparison with the gold standard translations and by exploratory triangulation using informal native-speaker discussions, web-based language resources, and LLMs.

### Ethical Considerations

This study was a methodological benchmarking analysis and did not involve human participants, patient-level data, or identifiable personal information. The materials analyzed consisted of EQ-5D-5L source-text segments and official, linguistically validated translations used as benchmark references for translation-quality evaluation. Because no human-participant research procedures were conducted and no identifiable human data were analyzed, institutional review board review was not required. Informed consent was not applicable. No compensation was provided. No identifying images of participants or users were included in the manuscript or supplementary materials.

## Results

### Overview of Analyses

The results are presented in 2 complementary parts. First, inferential analyses were used to test whether the 4 AI translation services differed from one another within each metric-language combination. Second, descriptive summaries and visual analyses were used to assess the distribution of translation quality scores relative to the validated human translations and to identify localized sentence-level hotspots.

### Statistical Comparison of AI Services

Translation quality scores from the 4 AI services (Google, GPT-4.1, Amazon, and DeepL) were compared across all 20 metric-language combinations. The Friedman tests revealed statistically significant differences in 11 of the 20 (55%) combinations, indicating that the performance distributions of the services were not identical.

To determine which specific services differed, paired Wilcoxon signed-rank post hoc tests with a Holm-Bonferroni correction were performed on the 11 significant Friedman test results. This analysis confirmed 11 statistically significant pairwise differences, with adjusted *P* values ranging from <.001 to .049. This is the central finding: the AI services are not functionally interchangeable, and statistically significant performance differences depended on both the target language and the evaluation metric.

### Descriptive and Inferential Findings With Effect Sizes

**Figure 2.**
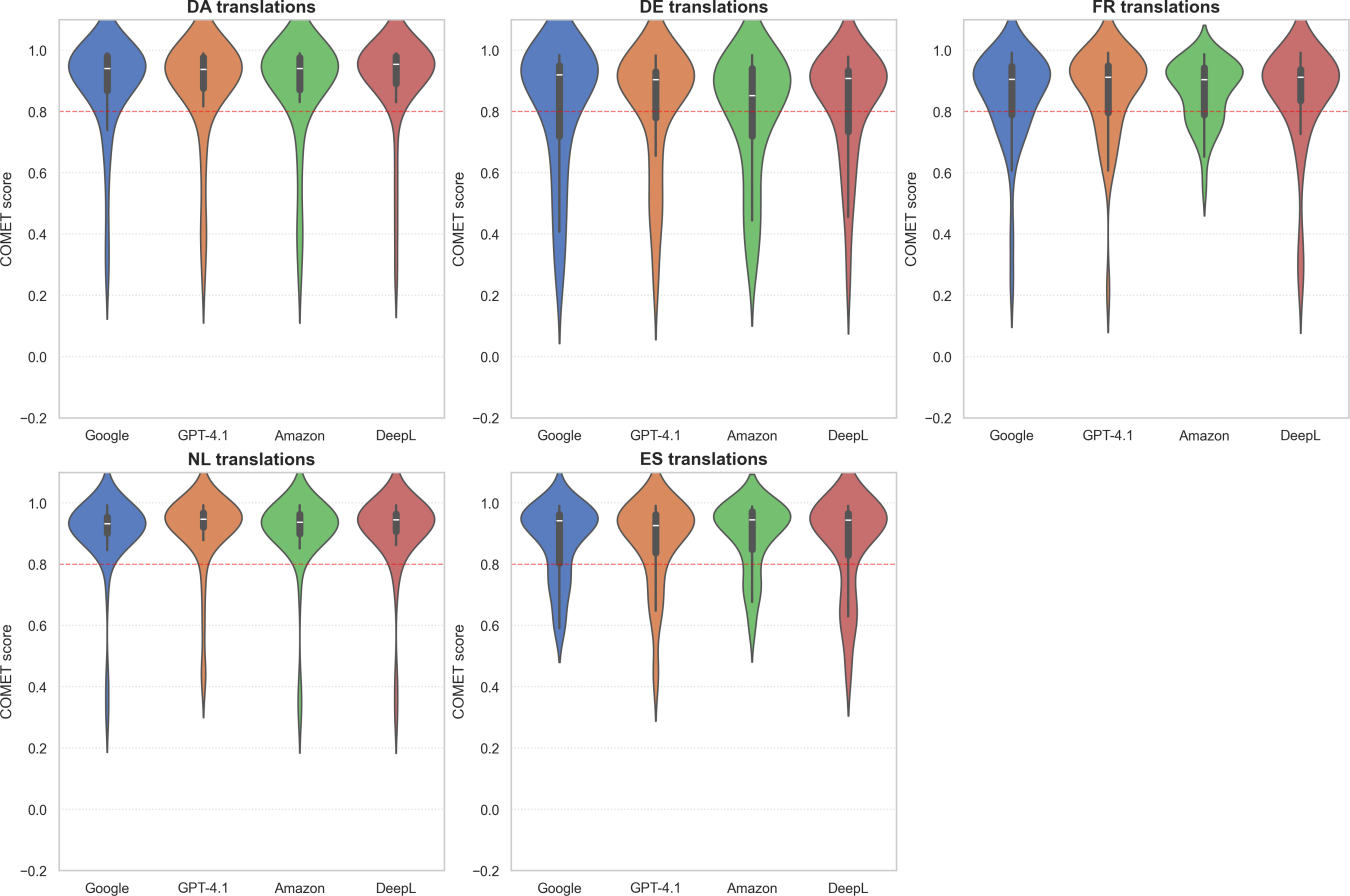
Distribution of sentence-level COMET scores across 5 target languages for 4 AI translation services in this methodological benchmarking study of EQ-5D-5L translation. Each violin plot summarizes the distribution of semantic similarity scores for the 43 translated source-text segments benchmarked against the official, linguistically validated human translations (gold standard). The dashed horizontal line at COMET=0.80 marks the descriptive threshold used to indicate strong semantic similarity. Across languages, most scores cluster above this threshold, consistent with generally high semantic comparability despite localized sentence-level variation. COMET: cross-lingual optimized metric for evaluation of translation. The target languages are represented in the figure as follows: DA: Danish; NL: Dutch; FR: French; DE: German; and ES: Spanish. EQ-5D-5L: EuroQol 5-dimension 5-level.

The significant differences clustered around specific metrics (Table 1). Of the 11 significant pairwise differences, 10 (91%) were identified by the surface-overlap metrics (BLEU and METEOR), which measure stylistic and lexical similarity. In contrast, the semantic metrics (COMET and BLEURT), which measure meaning, together only accounted for 1 of the 11 differences. Taken together, these findings suggest that the services differ more in stylistic realization than in meaning preservation. To visualize this semantic comparability directly, Figure 2 presents COMET score distributions, as COMET is a reference-based semantic metric with a strong reported correlation with human judgments. Individual score distributions for each target language are detailed in Multimedia Appendix 3.

The post hoc summary shows Holm-Bonferroni–adjusted *P* values and pairwise effect size *r* from paired Wilcoxon signed-rank tests for significant pairwise comparisons only. Friedman *P* values and post hoc adjusted *P* values address different levels of comparison and are, therefore, not directly comparable: the Friedman test evaluates an omnibus difference across all 4 services, whereas each Wilcoxon signed-rank test evaluates a specific service pair.

This interpretation was supported by the effect size analysis. Omnibus effect sizes were generally small, with a median Kendall *W* of 0.08 (IQR 0.07-0.10) across the 11 significant tests, indicating limited overall separation among all 4 AI translation services within a metric-language combination. In contrast, some post hoc pairwise effect sizes were moderate to large, showing that specific service pairs could differ meaningfully even when the overall omnibus separation remained modest.

For example, in the BLEU score for Spanish, Google (median 0.325, IQR 0.199-0.632) performed significantly worse than Amazon (median 0.425, IQR 0.230-1.000; adjusted *P*=.001; *r*=0.75). Conversely, for the semantic metric COMET in Dutch, the Friedman test indicated an overall difference among services, but no pairwise comparison remained significant after Holm-Bonferroni correction. This finding reinforces the interpretation that semantic metric differences were limited and that most significant pairwise differences were concentrated in surface-overlap metrics.

Threshold-based descriptive summaries further supported this interpretation. Across languages and services, most COMET and BLEURT scores fell within ranges consistent with high semantic similarity to the validated reference translations, whereas BLEU and METEOR showed a greater proportion of lower scores because of their sensitivity to lexical and word-order variation. Detailed threshold-based summaries by metric, language, and AI translation service are provided in Table S1 in Multimedia Appendix 3.

**Table 1.** Descriptive statistics and inferential comparison results for this methodological benchmarking study of AI-powered EQ-5D-5L translation^a^.

Metric	Language (code)	Chi-square (*df*); Friedman test	*P* value	Kendall *W*^b^	Google, median (IQR)	GPT-4.1, median (IQR)	Amazon, median (IQR)	DeepL, median (IQR)	Post hoc summary (adjusted *P* value; *r*)
COMET^c^	Danish (DA)	5.036 (3)	.17	0.04	0.941 (0.868-0.981)	0.938 (0.876-0.977)	0.941 (0.871-0.976)	0.954 (0.890-0.980)	No significant difference
COMET	German (DE)	8.118 (3)	.04	0.06	0.920 (0.720-0.949)	0.905 (0.779-0.930)	0.852 (0.720-0.941)	0.909 (0.734-0.933)	No pairwise differences after correction
COMET	French (FR)	6.01 (3)	.11	0.05	0.905 (0.789-0.947)	0.912 (0.796-0.950)	0.904 (0.789-0.942)	0.912 (0.836-0.936)	No significant difference
COMET	Dutch (NL)	9.296 (3)	.03	0.07	0.933 (0.901-0.954)	0.947 (0.921-0.968)	0.937 (0.899-0.963)	0.945 (0.907-0.964)	No pairwise differences after correction
COMET	Spanish (ES)	7.56 (3)	.06	0.06	0.943 (0.803-0.962)	0.926 (0.837-0.962)	0.945 (0.848-0.971)	0.944 (0.829-0.966)	No significant difference
BLEU^d^	Danish (DA)	1.11 (3)	.78	0.009	0.707 (0.294-1.000)	0.569 (0.301-1.000)	0.502 (0.230-0.867)	0.649 (0.230-1.000)	No significant difference
BLEU	German (DE)	10.478 (3)	.02	0.08	0.508 (0.147-0.760)	0.393 (0.129-0.702)	0.323 (0.086-0.695)	0.446 (0.162-0.718)	Google vs GPT-4.1 (adjusted *P*=.03; *r*=0.54)
BLEU	French (FR)	12.551 (3)	.006	0.10	0.102 (0.054-0.304)	0.222 (0.066-0.361)	0.095 (0.054-0.261)	0.161 (0.062-0.248)	GPT-4.1 vs Amazon (adjusted *P*=.004; *r*=0.66); Google vs GPT-4.1 (adjusted *P*=.01; *r*=0.62)
BLEU	Dutch (NL)	8.027 (3)	.047	0.06	0.382 (0.097-0.745)	0.597 (0.231-0.800)	0.322 (0.086-0.652)	0.395 (0.077-0.728)	GPT-4.1 vs Amazon (adjusted *P*=.02; *r*=0.63)
BLEU	Spanish (ES)	20.661 (3)	<.001	0.16	0.325 (0.199-0.632)	0.425 (0.175-0.743)	0.425 (0.230-1.000)	0.398 (0.240-0.688)	Google vs Amazon (adjusted *P*=.001; *r*=0.75)
METEOR^e^	Danish (DA)	2.122 (3)	.55	0.02	0.837 (0.642-0.998)	0.837 (0.658-0.998)	0.807 (0.639-0.944)	0.865 (0.653-0.998)	No significant difference
METEOR	German (DE)	9.684 (3)	.02	0.08	0.769 (0.490-0.984)	0.711 (0.509-0.830)	0.598 (0.354-0.830)	0.778 (0.522-0.830)	No pairwise differences after correction
METEOR	French (FR)	17.166 (3)	<.001	0.13	0.391 (0.264-0.591)	0.500 (0.347-0.753)	0.301 (0.223-0.516)	0.424 (0.343-0.517)	GPT-4.1 vs Amazon (adjusted *P*<.001; *r*=0.81); Google vs GPT-4.1 (adjusted *P*=.005; *r*=0.68); GPT-4.1 vs DeepL (adjusted *P*=.049; *r*=0.44)
METEOR	Dutch (NL)	11.305 (3)	.01	0.09	0.747 (0.571-0.869)	0.830 (0.692-0.981)	0.701 (0.562-0.843)	0.736 (0.625-0.882)	GPT-4.1 vs Amazon (adjusted *P*<.001; *r*=0.80); Google vs GPT-4.1 (adjusted *P*=.03; *r*=0.67)
METEOR	Spanish (ES)	11.795 (3)	.008	0.09	0.755 (0.708-0.963)	0.810 (0.724-0.976)	0.803 (0.661-0.996)	0.755 (0.628-0.981)	No pairwise differences after correction
BLEURT^f^	Danish (DA)	5.798 (3)	.12	0.05	0.890 (0.756-0.964)	0.910 (0.805-0.959)	0.875 (0.762-0.938)	0.904 (0.811-0.960)	No significant difference
BLEURT	German (DE)	4.409 (3)	.22	0.03	0.874 (0.756-0.914)	0.834 (0.718-0.875)	0.819 (0.765-0.888)	0.842 (0.777-0.874)	No significant difference
BLEURT	French (FR)	8.737 (3)	.03	0.07	0.827 (0.727-0.896)	0.853 (0.759-0.903)	0.831 (0.733-0.900)	0.829 (0.763-0.872)	Google vs GPT-4.1 (adjusted *P*=.04; *r*=0.53)
BLEURT	Dutch (NL)	3.807 (3)	.28	0.03	0.852 (0.802-0.964)	0.905 (0.830-0.957)	0.856 (0.790-0.931)	0.890 (0.802-0.968)	No significant difference
BLEURT	Spanish (ES)	4.846 (3)	.18	0.04	0.905 (0.855-0.942)	0.930 (0.841-0.967)	0.930 (0.818-0.993)	0.909 (.841-0.971)	No significant difference

a For each of the 20 metric-language combinations, the table reports sentence-level median and IQR (Q1-Q3) scores across 43 source-text segments for 4 AI services, together with Friedman test results, Kendall *W*, and Holm-adjusted Wilcoxon post hoc comparisons among AI services, based on scores computed against the gold standard translations.

b Kendall *W* values are individual omnibus effect sizes from the corresponding Friedman tests and are not median values. The median and IQR ranges apply only to the AI service score columns.

c COMET: cross-lingual optimized metric for evaluation of translation.

d BLEU: bilingual evaluation understudy.

e METEOR: metric for evaluation of translation with explicit ordering.

f BLEURT: bilingual evaluation understudy with representations from transformers.

### Qualitative Outlier Analysis

To supplement the aggregate analyses, we conducted a qualitative outlier analysis by benchmarking AI outputs directly against the official, linguistically validated human translations (gold standard). Outliers were defined as sentence-level scores at or below the 5th percentile for each metric. This analysis was intended to localize failure modes rather than determine whether translation services were globally acceptable or unacceptable.

As a descriptive count-based sensitivity analysis, BLEU was the most discriminative metric for flagging potential low-scoring segments (158 instances), followed by METEOR (73), BLEURT (60), and COMET (60). Outlier counts were then examined across services. The distribution was relatively even, ranging from 85 flagged instances for DeepL to 92 for Amazon Translate, suggesting that all services showed a similar frequency of localized deviations from the gold standard.

**Figure 3.**
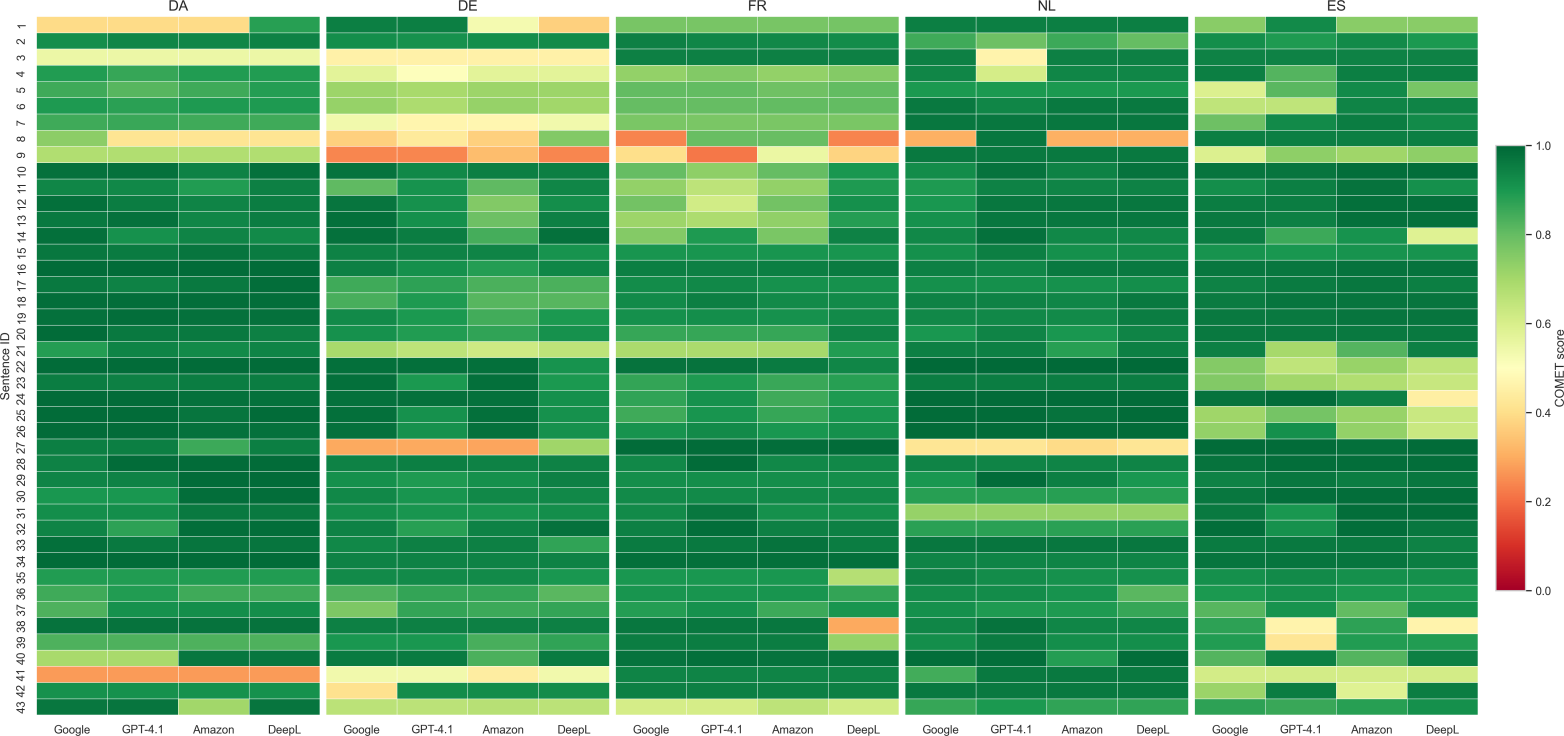
Sentence-level COMET heatmap across 5 target languages and 4 AI translation services in this methodological benchmarking study of EQ-5D-5L translation. Rows correspond to the 43 source segments and columns correspond to the AI services within each language panel. Darker green indicates higher semantic similarity to the official, linguistically validated human translation, whereas lighter colors indicate lower scores and localized translation hotspots. The figure illustrates that most segments scored highly across services, but lower-scoring deviations clustered in specific source segments rather than being randomly distributed across the instrument. See Multimedia Appendix 2 for the sentence-number mapping guide. COMET: cross-lingual optimized metric for evaluation of translation. The target languages are represented in the figure as follows: DA: Danish; NL: Dutch; FR: French; DE: German; and ES: Spanish. EQ-5D-5L: EuroQol 5-dimension 5-level.

Outliers were not randomly distributed but clustered within specific hotspots (Figure 3). Language-specific heatmaps are provided in Multimedia Appendix 3, whereas Multimedia Appendix 2 provides a sentence-number mapping guide for the 43 source segments. Because the EQ-5D-5L source instrument is proprietary, this mapping is presented as annotated screenshots from the sample UK English digital questionnaire in the EuroQol user guide rather than as a reprinted text table.

Some of these hotspots corresponded to short digital interface elements rather than clinical questionnaire constructs. In particular, the final source segments included navigation and interface strings such as “Previous,” “Next,” and an error message. These elements are context-dependent and may be difficult to translate optimally when evaluated as isolated sentence-level strings outside their software environment. Therefore, low scores for these segments should be interpreted separately from deviations involving clinical concepts, domain headers, or response options.

BLEU identified the largest hotspot clusters in Spanish and Dutch, indicating where review effort may need to be concentrated for specific language-service combinations. However, because hotspot counts alone do not establish overall adequacy, overall translation quality was interpreted based on the full score distributions and semantic metric patterns, and heatmaps were used specifically to localize low-scoring deviations.

To illustrate the clinical implications of these findings, we conducted a qualitative review of both high-scoring and low-scoring sentences (Table 2). For simple declarative sentences, the AI models frequently achieved perfect alignment with the human reference translations, producing identical, verbatim matches across all 4 services (eg, the German translation for pain severity). This confirms that, for standard grammatical structures and unambiguous phrasing, AI performance was highly reliable and consistent.

However, for section headers, abstract health concepts, and short context-dependent interface strings, some differences from the gold standard emerged. As detailed in Table 2, AI services sometimes defaulted to literal or highly medicalized terminology rather than to the intended clinical concept. This pattern was observed across languages; for instance, in Danish, models frequently selected terms that were technically correct but contextually distinct (eg, translating “Mobility” as the sociological concept *Mobilitet* rather than the physical capacity *Bevægelighed* used in the validated version). These examples illustrate a possible limitation of current AI models in distinguishing literal translation from conceptual equivalence. The practical relevance of isolated header differences could not be assessed in the present study. Further equivalence or cognitive-debriefing research will be needed to determine which discrepancies are acceptable and which require the involvement of expert human linguists. Therefore, Table 2 presents representative examples of both high-scoring exact matches and low-scoring conceptual deviations, while the heatmap and sentence-number mapping identify additional hotspots, including interface-related strings.

**Table 2.** Representative high-scoring and low-scoring examples from this methodological benchmarking study of AI-powered EuroQol 5-dimension 5-level (EQ-5D-5L) translation^a^.

Language (code)	Source text	Gold standard translation	AI translation (service)	Metric score	Error type	Qualitative error description
German (DE)	I have severe pain or discomfort	Ich habe starke Schmerzen oder Beschwerden	Ich habe starke Schmerzen oder Beschwerden (Google)	BLEU^b^: 1.0 METEOR^c^: 1.0 COMET^d^: 0.983 BLEURT^e^: 0.964	None	All 4 services produced an identical match to the human reference, demonstrating high reliability for simple declarative sentences.
Danish (DA)	The best health you can imagine	Det bedste helbred, du kan forestille dig	Det bedste helbred, du kan forestille dig (GPT-4.1)	BLEU: 1.0 METEOR: 1.0 COMET: 0.975 BLEURT: 1.0	None	All 4 services produced an identical match to the human reference, demonstrating high reliability for simple declarative sentences.
French (FR)	This scale is numbered from 0 to 100.	Cette échelle est numérotée de 0 à 100	Cette échelle est numérotée de 0 à 100 (Amazon)	BLEU: 1.0 METEOR: 1.0 COMET: 0.984 BLEURT: 0.968	None	All 4 services produced an identical match to the human reference, demonstrating high reliability for simple declarative sentences.
French (FR)	Self-care	AUTONOMIE DE LA PERSONNE	SOINS AUTO-ADMINISTRÉS (Google)	BLEU: 0.0 METEOR: 0.0 COMET: 0.398 BLEURT: 0.198	Conceptual	Literal translation implies “medical treatment” (administering care) rather than the clinical concept of “personal autonomy” (washing or dressing).
German (DE)	Pain or discomfort	SCHMERZEN / KÖRPERLICHE BESCHWERDEN	SCHMERZ / UNWOHLSEIN (Amazon)	BLEU: 0.0 METEOR: 0.0 COMET: 0.623 BLEURT: 0.445	Semantic	The AI translation “Unwohlsein” (feeling unwell or malaise) is a general subjective state, whereas the gold standard Beschwerden (complaints or discomfort) is a broader term encompassing specific physical pain or functional issues.
Dutch (NL)	Anxiety or depression	ANGST / SOMBERHEID	ANGST / DEPRESSIE (DeepL)	BLEU: 0.0 METEOR: 0.623 COMET: 0.419 BLEURT: 0.580	Intensity	The AI translation “Depressie” refers to a clinical psychiatric disorder (pathology), whereas the Gold Standard “Somberheid” captures the subjective feeling of gloom or sadness (symptom) required for general health reporting.
Danish (DA)	Mobility	BEVÆGELIGHED	MOBILITET (GPT-4.1)	BLEU: 0.0 METEOR: 0.0 COMET: 0.341 BLEURT: 0.271	Domain	The AI translation “Mobilitet” is used for sociology or transport (systemic movement), whereas the Gold Standard “Bevægelighed” refers to biological/physical capacity (range of motion).

a Examples are drawn from sentence-level comparisons between AI-generated translations and official linguistically validated human translations (gold standard) across 5 target languages and illustrate both exact matches and conceptually important deviations identified during hotspot analysis. Conceptual indicates incorrect rendering of the intended clinical construct; semantic indicates a meaning shift relative to the gold standard; intensity indicates altered severity or strength; and domain indicates a shift into an inappropriate usage context or domain.

b BLEU: bilingual evaluation understudy.

c METEOR: metric for evaluation of translation with explicit ordering.

d COMET: cross-lingual optimized metric for evaluation of translation.

e BLEURT: bilingual evaluation understudy with representations from transformers.

## Discussion

### Principal Findings

This study reveals that although AI translation services are not statistically identical, most between-service differences were stylistic rather than semantic. We identified 11 statistically significant pairwise differences (adjusted *P*<.05, ranging from <.001 to .049), of which 91% (10 of 11) were detected by surface-overlap metrics (BLEU/METEOR), whereas only 1 was detected by a semantic metric (BLEURT). Supported by the generally small omnibus effect sizes (median Kendall *W*=0.08, IQR 0.07-0.10), this pattern suggests that although services differed in lexical choice, they produced translations with highly comparable semantic meaning relative to the validated human translations. Taken together, the semantic score distributions and the localized nature of the low-scoring hotspots suggest that current AI services can generate translations for this instrument in the evaluated languages. However, this interpretation depends on the combined descriptive, semantic, and hotspot analyses rather than on significance testing alone. Some differences occurred in parts of the questionnaire outside the scored response options; however, their practical relevance for participant interpretation could not be assessed in the present study. Further research will be needed to determine which PROM elements (ie, instructions and answer options) should be focused on when evaluating translation quality.

### Defining the Human-in-the-Loop

The finding of statistical comparability of average scores must be interpreted with caution, as aggregate metrics can mask critical, low-incidence discrepancies. Our qualitative outlier analysis revealed that deviations from the gold standard were evenly distributed across all services (85‐92 outliers per service). The lowest-scoring deviations were primarily observed in domain headers, and the 4 representative low-scoring examples in Table 2 came from different AI services. These findings suggest that human oversight may be required across services, although further research is needed to determine the level of review required to ensure acceptable translation quality.

The potential clinical significance of these deviations was evaluated through a manual review of systemic inconsistencies against the official, linguistically validated translations. As detailed in Table 2, semantic inconsistencies occurred primarily in abstract concepts and domain headers rather than in the actual response options themselves. For instance, the domain header concept “Self-care” was frequently translated literally (eg, Soins auto-administrés or “self-administered medical care”) rather than conceptually (referring to personal autonomy in washing or dressing). Although the subsequent item text (“I have no problems washing or dressing”) was often translated correctly, the practical relevance of isolated header differences could not be assessed in the present study. This interpretation is consistent with electronic clinical outcome assessment migration literature, which distinguishes core respondent-facing content, such as instructions, item wording, and response options, from other contextual or presentation elements when considering the level of equivalence evidence required [2]. A follow-up study is planned to quantify the reduction in effort that AI can offer compared with fully manual translation.

### Limitations

We acknowledge several limitations. First, cultural adaptation was assessed only indirectly, as no patient cognitive debriefing was performed. Second, the study used sentence-level translation and evaluation to support aligned benchmarking against the gold standard, which may have constrained systems that benefit from broader document context and may not have fully captured dependence among related instrument segments. Third, the findings are limited to high-resource European languages and should not be generalized to lower-resource or structurally distinct languages without further validation. Fourth, the primary semantic metrics were reference-based and may therefore have penalized acceptable alternative phrasings that differed from the validated reference wording. Fifth, the service configuration was not fully symmetrical because GPT-4.1 was used with a structured medical translation prompt, whereas Google Translate, Amazon Translate, and DeepL were evaluated in their standard API mode. Sixth, because the EQ-5D-5L and its validated translations are widely used, some systems may have been exposed to similar wording during training or optimization. Finally, the qualitative error categories were interpretive and descriptive rather than based on a formally validated annotation framework, and no blinded, independent, multirater review or formal interrater reliability testing was performed.

### Future Research Directions

Future work will extend this benchmarking approach in several directions. Automated metric results should first be compared directly with expert human judgments to determine how well these scores reflect professional linguistic review of PROM translations. Studies should also quantify the operational value of human-in-the-loop workflows by comparing fully manual translation with AI-assisted translation followed by expert editing. Broader evaluation designs should examine whether performance changes when related questionnaire elements are analyzed at the domain level rather than strictly at the sentence level, and whether these findings generalize to structurally different or lower-resource languages. Finally, future studies should explore hybrid and reference-free evaluation strategies, including quality estimation methods, and assess how such approaches could be integrated into clinical trial translation workflows more effectively.

### Conclusions

This benchmarking study showed that leading AI translation services were not functionally interchangeable across metric-language combinations. Although statistically significant differences were identified, these were more often detected by surface-overlap metrics than by semantic metrics. These findings support the use of AI to generate PROM translations for this instrument and the evaluated high-resource European languages. Expert human linguist review may need to be retained to confirm conceptual equivalence and fitness for clinical use.

## Supplementary material

10.2196/78485 Multimedia Appendix 1 System prompt and reproducibility parameters for the GPT-4.1 model.

10.2196/78485 Multimedia Appendix 2 Annotated sentence-number mapping guide for the 43 EQ-5D-5L source segments based on the sample UK English digital questionnaire from the official EuroQol user guide.

10.2196/78485 Multimedia Appendix 3 Supplementary violin plots, heatmaps, and threshold-based descriptive summaries of sentence-level metric scores for each target language.

## Abbreviations

API: application programming interface
AWS: Amazon Web Services
BLEU: bilingual evaluation understudy
BLEURT: bilingual evaluation understudy with representations from transformers
COMET: cross-lingual optimized metric for evaluation of translation
EQ-5D-5L: EuroQol 5-dimension 5-level
LLM: large language model
METEOR: metric for evaluation of translation with explicit ordering
NMT: neural machine translation
PROM: patient-reported outcome measure

## References

[1] Acquadro C, Conway K, Girourdet C, Mear I (2004). Linguistic Validation Manual for Patient-Reported Outcomes (PRO) Instruments.

[2] Byrom B, Gwaltney C, Slagle A, Gnanasakthy A, Muehlhausen W (2019). Measurement equivalence of patient-reported outcome measures migrated to electronic formats: a review of evidence and recommendations for clinical trials and bring your own device. Ther Innov Regul Sci.

[3] Eremenco SL, Cella D, Arnold BJ (2005). A comprehensive method for the translation and cross-cultural validation of health status questionnaires. Eval Health Prof.

[4] Wild D, Grove A, Martin M (2005). Principles of good practice for the translation and cultural adaptation process for patient-reported outcomes (PRO) measures: report of the ISPOR Task Force for Translation and Cultural Adaptation. Value Health.

[5] Patrick DL, Burke LB, Gwaltney CJ (2011). Content validity—establishing and reporting the evidence in newly developed patient-reported outcomes (PRO) instruments for medical product evaluation: ISPOR PRO Good Research Practices Task Force report: part 2—assessing respondent understanding. Value Health.

[6] Harkness JA, Villar A, Edwards B, Harkness JA, Braun M, Edwards B, Johnson TP, Lyberg L, Mohler PP, Smith TW (2010). Survey Methods in Multinational, Multiregional, and Multicultural Contexts.

[7] Foote HP, Hong C, Anwar M (2025). Embracing generative artificial intelligence in clinical research and beyond: opportunities, challenges, and solutions. JACC Adv.

[8] van Kolfschooten H, Goosen S, van Oirschot J, Schouten B, Vajda I, Willems L (2025). Legal, ethical, and policy challenges of artificial intelligence translation tools in healthcare. Discov Public Health.

[9] Johnson M, Schuster M, Le QV (2017). Google's multilingual neural machine translation system: enabling zero-shot translation. Trans Assoc Comput Linguist.

[10] Wu Y, Schuster M, Chen Z (2016). Google’s neural machine translation system: bridging the gap between human and machine translation. arXiv.

[11] Solomou T, Mappouras S, Kyriacou E (2025). Bridging language barriers in healthcare: a patient-centric mobile app for multilingual health record access and sharing. Front Digit Health.

[12] Nguyen HH, Mahajan K, Yadav V Prompting with phonemes: enhancing llms’ multilinguality for non-latin script languages.

[13] Lu SC, Xu C, Kaur M, Edelen MO, Pusic A, Gibbons C (2025). Can machine translation match human expertise? Quantifying the performance of large language models in the translation of patient-reported outcome measures (PROMs). J Patient Rep Outcomes.

[14] Kwong JCC, Wang SCY, Nickel GC, Cacciamani GE, Kvedar JC (2024). The long but necessary road to responsible use of large language models in healthcare research. NPJ Digit Med.

[15] Quickstart. DeepL API documentation.

[16] Amazon Translate documentation. Amazon Web Services.

[17] Models. OpenAI Developers.

[18] Ataman D, Birch A, Habash N, Federico M, Koehn P, Cho K (2025). Machine translation in the era of large language models: a survey of historical and emerging problems. Information.

[19] Herdman M, Gudex C, Lloyd A (2011). Development and preliminary testing of the new five-level version of EQ-5D (EQ-5D-5L). Qual Life Res.

[20] (2019). EQ-5D-5L User Guide Version 3.0. https://euroqol.org/wp-content/uploads/2023/11/EQ-5D-5LUserguide-23-07.pdf.

[21] Janssen MF, Pickard AS, Golicki D (2013). Measurement properties of the EQ-5D-5L compared to the EQ-5D-3L across eight patient groups: a multi-country study. Qual Life Res.

[22] Papineni K, Roukos S, Ward T, Zhu WJ, Isabelle P, Charniak E, Lin D (2002). Proceedings of the 40th Annual Meeting of the Association for Computational Linguistics.

[23] Banerjee S, Lavie A, Goldstein J, Lavie A, Lin CY, Voss C (2005). Proceedings of the ACL Workshop on Intrinsic and Extrinsic Evaluation Measures for Machine Translation and/or Summarization.

[24] Rei R, Stewart C, Farinha AC, Lavie A, Webber B, Cohn T, He Y, Liu Y (2020). Proceedings of the 2020 Conference on Empirical Methods in Natural Language Processing.

[25] Sellam T, Das D, Parikh A, Jurafsky D, Chai J, Schluter N, Tetreault J (2020). Proceedings of the 58th Annual Meeting of the Association for Computational Linguistics.

[26] Guerreiro NM, Rei R, van Stigt D, Coheur L, Colombo P, Martins AFT (2024). xCOMET: transparent machine translation evaluation through fine-grained error detection. Trans Assoc Comput Linguist.

