# Supplementary material for: Benchmarking AI-Powered Translation of the EQ-5D-5L Patient-Reported Outcome Measure Using Automated Metrics: Comparative Evaluation Study
Source: JMIR AI. 2026 Aug 4;5:e78485. doi: 10.2196/78485 (PMC13436651; doi:10.2196/78485)
Supplement: Supplementary file 2 [file ai-v5-e78485-s002.docx]

**Multimedia Appendix 2: Sentence-number mapping guide for the 43 EQ-5D-5L source segments based on the sample UK English digital version from the EuroQol User Guide.**

This appendix provides a structural mapping aid for the 43 source segments analyzed in the sentence-level heatmaps. Because the EQ-5D-5L instrument is proprietary, the mapping is presented as annotated screenshots of the sample UK English digital version from the official EuroQol User Guide rather than as a reprinted text table.

This appendix is provided for segment numbering and visualization only and does not alter the underlying source-segment set used in the analysis.

Legend: Numbers in RED indicate the sentence/segment identifiers used in the manuscript heatmaps.
