# Supplementary material for: Benchmarking AI-Powered Translation of the EQ-5D-5L Patient-Reported Outcome Measure Using Automated Metrics: Comparative Evaluation Study
Source: JMIR AI. 2026 Aug 4;5:e78485. doi: 10.2196/78485 (PMC13436651; doi:10.2196/78485)
Supplement: Supplementary file 3 [file ai-v5-e78485-s003.docx]

## **Multimedia Appendix 3: Supplementary language-specific plots, heatmaps, and threshold-based descriptive summaries of sentence-level translation quality scores**

### **Danish (DA)**

**Supplementary Figure S1:** Distribution of semantic quality (COMET) scores for Danish translations.

**Supplementary Figure S2:** Sentence-level difficulty heatmap (COMET scores) for Danish (DA) translations.

### **German (DE)**

**Supplementary Figure S3:** Distribution of semantic quality (COMET) scores for German translations.

**Supplementary Figure S4:** Sentence-level difficulty heatmap (COMET scores) for German (DE) translations.

**French (FR)**

**Supplementary Figure S5:** Distribution of semantic quality (COMET) scores for French translations.

**Supplementary Figure S6:** Sentence-level difficulty heatmap (COMET scores) for French (FR) translations.

**Dutch (NL)**

**Supplementary Figure S7:** Distribution of semantic quality (COMET) scores for Dutch translations.

**Supplementary Figure S8:** Sentence-level difficulty heatmap (COMET scores) for Dutch (NL) translations.

**Spanish (ES)**

**Supplementary Figure S9:** Distribution of semantic quality (COMET) scores for Spanish translations.

**Supplementary Figure S10:** Sentence-level difficulty heatmap (COMET scores)for Spanish (ES) translations.

**Supplementary Table S1.** Proportion of sentence-level scores within and below descriptive quality bands by metric, language, and AI service.

| **Metric** | **Language** | **AI service** | **High band n/N (%)** | **Mid band n/N (%)** | **Low band n/N (%)** |
| --- | --- | --- | --- | --- | --- |
| BLEU | Danish | Google | 24/43 (55.8%) | 8/43 (18.6%) | 11/43 (25.6%) |
| BLEU | Danish | GPT-4.1 | 23/43 (53.5%) | 9/43 (20.9%) | 11/43 (25.6%) |
| BLEU | Danish | Amazon | 22/43 (51.2%) | 8/43 (18.6%) | 13/43 (30.2%) |
| BLEU | Danish | DeepL | 24/43 (55.8%) | 5/43 (11.6%) | 14/43 (32.6%) |
| BLEU | German | Google | 23/43 (53.5%) | 2/43 (4.7%) | 18/43 (41.9%) |
| BLEU | German | GPT-4.1 | 16/43 (37.2%) | 9/43 (20.9%) | 18/43 (41.9%) |
| BLEU | German | Amazon | 18/43 (41.9%) | 4/43 (9.3%) | 21/43 (48.8%) |
| BLEU | German | DeepL | 21/43 (48.8%) | 7/43 (16.3%) | 15/43 (34.9%) |
| BLEU | Spanish | Google | 14/43 (32.6%) | 10/43 (23.3%) | 19/43 (44.2%) |
| BLEU | Spanish | GPT-4.1 | 16/43 (37.2%) | 10/43 (23.3%) | 17/43 (39.5%) |
| BLEU | Spanish | Amazon | 21/43 (48.8%) | 6/43 (14.0%) | 16/43 (37.2%) |
| BLEU | Spanish | DeepL | 12/43 (27.9%) | 13/43 (30.2%) | 18/43 (41.9%) |
| BLEU | French | Google | 6/43 (14.0%) | 6/43 (14.0%) | 31/43 (72.1%) |
| BLEU | French | GPT-4.1 | 9/43 (20.9%) | 8/43 (18.6%) | 26/43 (60.5%) |
| BLEU | French | Amazon | 6/43 (14.0%) | 4/43 (9.3%) | 33/43 (76.7%) |
| BLEU | French | DeepL | 2/43 (4.7%) | 3/43 (7.0%) | 38/43 (88.4%) |
| BLEU | Dutch | Google | 16/43 (37.2%) | 10/43 (23.3%) | 17/43 (39.5%) |
| BLEU | Dutch | GPT-4.1 | 23/43 (53.5%) | 8/43 (18.6%) | 12/43 (27.9%) |
| BLEU | Dutch | Amazon | 14/43 (32.6%) | 11/43 (25.6%) | 18/43 (41.9%) |
| BLEU | Dutch | DeepL | 17/43 (39.5%) | 9/43 (20.9%) | 17/43 (39.5%) |
| METEOR | Danish | Google | 31/43 (72.1%) | 8/43 (18.6%) | 4/43 (9.3%) |
| METEOR | Danish | GPT-4.1 | 31/43 (72.1%) | 7/43 (16.3%) | 5/43 (11.6%) |
| METEOR | Danish | Amazon | 31/43 (72.1%) | 4/43 (9.3%) | 8/43 (18.6%) |
| METEOR | Danish | DeepL | 30/43 (69.8%) | 8/43 (18.6%) | 5/43 (11.6%) |
| METEOR | German | Google | 26/43 (60.5%) | 10/43 (23.3%) | 7/43 (16.3%) |
| METEOR | German | GPT-4.1 | 22/43 (51.2%) | 14/43 (32.6%) | 7/43 (16.3%) |
| METEOR | German | Amazon | 18/43 (41.9%) | 13/43 (30.2%) | 12/43 (27.9%) |
| METEOR | German | DeepL | 27/43 (62.8%) | 8/43 (18.6%) | 8/43 (18.6%) |
| METEOR | Spanish | Google | 32/43 (74.4%) | 7/43 (16.3%) | 4/43 (9.3%) |
| METEOR | Spanish | GPT-4.1 | 33/43 (76.7%) | 7/43 (16.3%) | 3/43 (7.0%) |
| METEOR | Spanish | Amazon | 31/43 (72.1%) | 6/43 (14.0%) | 6/43 (14.0%) |
| METEOR | Spanish | DeepL | 29/43 (67.4%) | 11/43 (25.6%) | 3/43 (7.0%) |
| METEOR | French | Google | 8/43 (18.6%) | 12/43 (27.9%) | 23/43 (53.5%) |
| METEOR | French | GPT-4.1 | 11/43 (25.6%) | 16/43 (37.2%) | 16/43 (37.2%) |
| METEOR | French | Amazon | 6/43 (14.0%) | 12/43 (27.9%) | 25/43 (58.1%) |
| METEOR | French | DeepL | 3/43 (7.0%) | 21/43 (48.8%) | 19/43 (44.2%) |
| METEOR | Dutch | Google | 28/43 (65.1%) | 12/43 (27.9%) | 3/43 (7.0%) |
| METEOR | Dutch | GPT-4.1 | 32/43 (74.4%) | 11/43 (25.6%) | 0/43 (0.0%) |
| METEOR | Dutch | Amazon | 22/43 (51.2%) | 16/43 (37.2%) | 5/43 (11.6%) |
| METEOR | Dutch | DeepL | 26/43 (60.5%) | 14/43 (32.6%) | 3/43 (7.0%) |
| COMET | Danish | Google | 37/43 (86.0%) | 3/43 (7.0%) | 3/43 (7.0%) |
| COMET | Danish | GPT-4.1 | 37/43 (86.0%) | 2/43 (4.7%) | 4/43 (9.3%) |
| COMET | Danish | Amazon | 37/43 (86.0%) | 2/43 (4.7%) | 4/43 (9.3%) |
| COMET | Danish | DeepL | 39/43 (90.7%) | 1/43 (2.3%) | 3/43 (7.0%) |
| COMET | German | Google | 30/43 (69.8%) | 5/43 (11.6%) | 8/43 (18.6%) |
| COMET | German | GPT-4.1 | 32/43 (74.4%) | 4/43 (9.3%) | 7/43 (16.3%) |
| COMET | German | Amazon | 29/43 (67.4%) | 6/43 (14.0%) | 8/43 (18.6%) |
| COMET | German | DeepL | 31/43 (72.1%) | 6/43 (14.0%) | 6/43 (14.0%) |
| COMET | Spanish | Google | 32/43 (74.4%) | 9/43 (20.9%) | 2/43 (4.7%) |
| COMET | Spanish | GPT-4.1 | 34/43 (79.1%) | 7/43 (16.3%) | 2/43 (4.7%) |
| COMET | Spanish | Amazon | 35/43 (81.4%) | 7/43 (16.3%) | 1/43 (2.3%) |
| COMET | Spanish | DeepL | 32/43 (74.4%) | 8/43 (18.6%) | 3/43 (7.0%) |
| COMET | French | Google | 30/43 (69.8%) | 11/43 (25.6%) | 2/43 (4.7%) |
| COMET | French | GPT-4.1 | 31/43 (72.1%) | 11/43 (25.6%) | 1/43 (2.3%) |
| COMET | French | Amazon | 30/43 (69.8%) | 12/43 (27.9%) | 1/43 (2.3%) |
| COMET | French | DeepL | 34/43 (79.1%) | 6/43 (14.0%) | 3/43 (7.0%) |
| COMET | Dutch | Google | 40/43 (93.0%) | 1/43 (2.3%) | 2/43 (4.7%) |
| COMET | Dutch | GPT-4.1 | 38/43 (88.4%) | 3/43 (7.0%) | 2/43 (4.7%) |
| COMET | Dutch | Amazon | 40/43 (93.0%) | 1/43 (2.3%) | 2/43 (4.7%) |
| COMET | Dutch | DeepL | 39/43 (90.7%) | 2/43 (4.7%) | 2/43 (4.7%) |
| BLEURT | Danish | Google | 37/43 (86.0%) | 4/43 (9.3%) | 2/43 (4.7%) |
| BLEURT | Danish | GPT-4.1 | 39/43 (90.7%) | 2/43 (4.7%) | 2/43 (4.7%) |
| BLEURT | Danish | Amazon | 38/43 (88.4%) | 3/43 (7.0%) | 2/43 (4.7%) |
| BLEURT | Danish | DeepL | 38/43 (88.4%) | 3/43 (7.0%) | 2/43 (4.7%) |
| BLEURT | German | Google | 33/43 (76.7%) | 7/43 (16.3%) | 3/43 (7.0%) |
| BLEURT | German | GPT-4.1 | 32/43 (74.4%) | 9/43 (20.9%) | 2/43 (4.7%) |
| BLEURT | German | Amazon | 34/43 (79.1%) | 7/43 (16.3%) | 2/43 (4.7%) |
| BLEURT | German | DeepL | 36/43 (83.7%) | 5/43 (11.6%) | 2/43 (4.7%) |
| BLEURT | Spanish | Google | 40/43 (93.0%) | 2/43 (4.7%) | 1/43 (2.3%) |
| BLEURT | Spanish | GPT-4.1 | 39/43 (90.7%) | 4/43 (9.3%) | 0/43 (0.0%) |
| BLEURT | Spanish | Amazon | 38/43 (88.4%) | 5/43 (11.6%) | 0/43 (0.0%) |
| BLEURT | Spanish | DeepL | 41/43 (95.3%) | 2/43 (4.7%) | 0/43 (0.0%) |
| BLEURT | French | Google | 35/43 (81.4%) | 6/43 (14.0%) | 2/43 (4.7%) |
| BLEURT | French | GPT-4.1 | 38/43 (88.4%) | 3/43 (7.0%) | 2/43 (4.7%) |
| BLEURT | French | Amazon | 36/43 (83.7%) | 5/43 (11.6%) | 2/43 (4.7%) |
| BLEURT | French | DeepL | 38/43 (88.4%) | 3/43 (7.0%) | 2/43 (4.7%) |
| BLEURT | Dutch | Google | 42/43 (97.7%) | 1/43 (2.3%) | 0/43 (0.0%) |
| BLEURT | Dutch | GPT-4.1 | 39/43 (90.7%) | 4/43 (9.3%) | 0/43 (0.0%) |
| BLEURT | Dutch | Amazon | 42/43 (97.7%) | 1/43 (2.3%) | 0/43 (0.0%) |
| BLEURT | Dutch | DeepL | 40/43 (93.0%) | 3/43 (7.0%) | 0/43 (0.0%) |

Note: Quality bands are descriptive and are provided to aid interpretation of score distributions. They are pragmatic interpretive bands rather than formally validated pass/fail cutoffs for PROM translation quality. For BLEU and METEOR, high was defined as ≥0.50, mid as 0.30 to <0.50, and low as <0.30. For COMET and BLEURT, high was defined as ≥0.80, mid as 0.60 to <0.80, and low as <0.60. Abbreviation: n/N = number of sentence-level scores within a band / total number of sentence-level scores for that metric-language-service combination.
